# Proteinuria in systemic sclerosis: reversal by ACE inhibition

**DOI:** 10.1007/s00296-013-2691-6

**Published:** 2013-02-28

**Authors:** J. Schuster, P. Moinzadeh, C. Kurschat, T. Benzing, T. Krieg, M. Weber, N. Hunzelmann

**Affiliations:** 1 Department of Dermatology, University of Cologne, Cologne, Germany; 2 Renal Division, Department of Medicine and Center for Molecular Medicine, University of Cologne, Cologne, Germany; 3 Cologne Excellence Cluster on Cellular Stress Responses in Aging-Associated Diseases, University of Cologne, Cologne, Germany; 4 Department of Internal Medicine I, Merheim Medical Center, Cologne General Hospital, University Witten-Herdecke, Witten, Germany

**Keywords:** Systemic sclerosis, Proteinuria, Urine microelectrophoresis, ACE inhibitor therapy

## Abstract

In systemic sclerosis (SSc), kidney damage is a major clinical problem which can lead to a deleterious outcome. Recently, in diabetes mellitus, early detection of proteinuria and treatment with angiotensin-converting enzyme (ACE) inhibitors has been shown to slow progression of kidney disease and to improve prognosis. In this study, we investigated the spontaneous course of proteinuria in SSc and the effects of ACE inhibitor therapy. Proteinuria was determined in SSc patients with urine protein electrophoresis. SSc patients with proteinuria (*n* = 31) were followed over a median of 12 months. Of all 31 patients with pathologic urine protein electrophoresis investigated in this study, 9 patients (29 %) had additional microalbuminuria and 4 patients (12.9 %) showed increased total urinary protein. ACE inhibitor treatment was subsequently given to 23 patients. A total of 8 patients remained untreated for various reasons. Proteinuria resolved in 74 % of patients treated with ACE inhibitors, whereas in the untreated group, remission was observed only in 25 % (*p* = 0.014). Improvement of proteinuria was predominantly achieved in patients with recently diagnosed proteinuria and short disease duration. In patients with SSc and proteinuria, initiation of ACE inhibitor therapy resulted in a significant decrease in proteinuria.

## Introduction

Systemic sclerosis (SSc) is characterized by progressive involvement of the vascular system resulting in impairment of the affected organs. Involvement of kidney arteries manifesting as renal crisis is a well-known, acute, life-threatening complication. In autopsy studies, the incidence of renal disease on a histopathological level was reported to be 70–90 % [1], whereas Steen et al. [2] reported up to 32 % of renal involvement by clinical assessment of proteinuria and renal function. In recent epidemiological studies, the frequency of the most severe form of kidney involvement, scleroderma renal crisis, is probably below 5 % [3–7, unpublished observation, Hunzelmann N.].

However, the impact of SSc-mediated chronic vascular damage on glomerular integrity has not been examined in detail yet. In diabetic patients, damage of the glomerular capillaries leading to microalbuminuria and overt proteinuria is a well-known phenomenon. Vascular kidney damage can be detected at an early stage by increased permeability for proteins passing the glomerular filtration barrier [8, 9]. Various studies have dealt with the relationship between microalbuminuria and blood vessel damage in different populations. These studies were able to show that microalbuminuria can be used as an early marker for cardiovascular as well as non-cardiovascular mortality [10–14]. Interestingly, in SSc, two recent epidemiological studies identified proteinuria also as a risk factor for increased mortality [15, 16].

A number of well-controlled studies have shown that ACE inhibitors are capable of reducing proteinuria and of stabilizing renal function in diabetic and non-diabetic nephropathy [17–19]. Therefore, the aim of this study was to investigate whether ACE inhibitor therapy improves proteinuria in SSc patients.

## Materials and methods

In this study, we investigated 31 clinically well-characterized SSc patients with pathological urine microelectrophoresis. The study was regarded as exempt research without requirement for informed consent as this was review of existing data. Detailed patient characteristics are shown in Table 1. Clinical data of patients were obtained via a disease- and organ-specific questionnaire of the German Network for Systemic Sclerosis (DNSS) as previously published [3]. All patients fulfilled the American College of Rheumatology (ACR) criteria for SSc classification and were categorized as diffuse, limited or overlap syndrome as previously described [3]. Diabetes mellitus, cardiac disease (palpitations, conduction disturbances and diastolic dysfunction), arterial hypertension, cardiovascular disease and pre-existing renal disease, defined as the presence of renal insufficiency encompassing insufficiency due to acute renal crisis (creatinine clearance <80 ml/min × 1.73 m^2^ of a 24-h urine collection, proteinuria or albuminuria), were documented as relevant concomitant diseases. Blood chemistry and serological tests included the measurement of serum creatinine and autoantibody levels. Six patients had hypertension, none of the patients had diabetes mellitus, 6 had a smoking history, and 2 were active smokers. Patients were followed over a median period of 12 months (range 3–18 months).

**Table 1:** Patient characteristics

Gender, age	
Male	16.1 % (5/31)
Female	83.9 % (26/31)
Age (average/range in years)	62.09 % (41–74)
SSc subtypes	
lcSSc	54.8 % (17/31)
dcSSc	35.5 % (11/31)
Overlap	9.7 % (3/31)
Antibody status	
ACA	51.6 % (16/31)
Scl-70	29.0 % (9/31)
Organ involvement	
Pulmonary arterial hypertension (PAH)	19.4 % (6/31)
Lung fibrosis	35.5 % (11/31)
Cardiac disease	19.4 % (6/31)
Cardiovascular disease	6.4 % (2/31)
GI	74.2 % (23/31)
Musculoskeletal	22.6 % (7/31)
Pre-existing renal disease	9.7 % (3/31)
Organ symptoms and laboratory parameters	
mRSS <12	74.2 % (23/31)
mRSS ≥12	25.8 % (8/31)
Increased serum creatinine	3.2 % (1/31)
Increased urea	6.4 % (2/31)
ESR <21 mm/h	50.0 % (13/26)
ESR ≥21 mm/h	50.0 % (13/26)
Digital ulcers	25.8 % (8/31)
Hypertension	19.4 % (6/31)
Diabetes mellitus	0 % (0/31)
Systemic therapy	
Steroids	29.0 % (9/31)
Immunosuppressive therapy	19.4 % (6/31)

Urine samples of a 24-h urine collection were tested for albumin (Bromocresol green (BCG)-plus method), total protein (benzethonium chloride method) and creatinine (j3-Aminoghenazon (PAP) method). Pathologic albuminuria was defined as ≥30 mg/24 h (male) or ≥20 mg/24 h (female) or ≥25 mg/g urine creatinine (male) and ≥17 mg/g urine creatinine (female). Pathological total proteinuria was defined as followed: ≥300 mg/24 h or ≥200 mg/l or ≥200 mg/g urine creatinine.

**Fig. 1:**
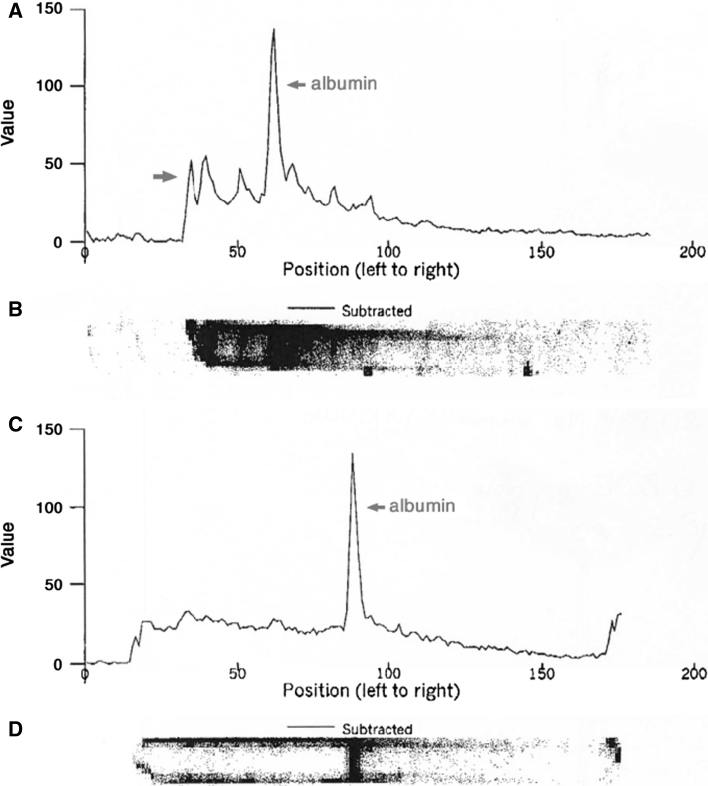
Urine microelectrophoresis before and after ACE inhibitor treatment A typical scan profile of urinary microelectrophoresis before **a** and after **c** ACE inhibitor treatment is shown (Pat. 5, Fig. 2). The area under the curves reflects total proteinuria. *Arrows* indicate the position of albumin, *x*-axis represents molecular weight (kD) and *y*-axis absorbance of the scanning procedure. The numbers are arbitrary units of the scanning procedure. The respective Coomassie blue stained gel is shown in **b** and **d**

In addition, a more detailed qualitative examination of excreted proteins was performed by urine microelectrophoresis (Fig. 1), which is about 70× more sensitive than conventional dip stick analysis. This technique uses undiluted urine samples, which are analysed by polyacrylamide gradient microgel electrophoresis, subsequent Coomassie blue staining and densitometric scanning. It allows the sharp separation and detection of urine proteins within a range of 10–500 kD. The results of each patient were graded either as normal, low-molecular-weight proteinuria, intermediate-molecular-weight proteinuria or as high-molecular-weight proteinuria as previously described [20].

Patient data were analysed with reference to skin, organ involvement and concomitant medication and their correlation with total urine protein excretion and urine protein composition.

Angiotensin-converting enzyme (ACE) inhibitors treatment with ramipril 2.5 or 5 mg had been started in 23 patients as a preventive measure at the discretion of the treating physician. Eight patients did not receive ACE inhibitor treatment due to distinctive hypotension, known incompatibilities or refusal by the patient. Consequently, these patients could be used in the control group and were shown to match in sex, age and ethnicity with the treated group by Mann–Whitney *U* test (data not shown).

Statistical analysis was performed using SPSS (statistical package for social sciences; version 14) with Pearson’s *χ*-test, Fisher’s exact test for dichotomous variables and the Mann–Whitney *U* test for nonparametric comparisons.

## Results

All of the 31 patients investigated had abnormal urinary low- and intermediate-molecular-weight protein excretion as determined by microelectrophoresis. A typical pathologic intermediate-molecular-weight proteinuria as densitometric printout is shown in Fig. 1. Additional microalbuminuria was detected in 9 (29.0 %) of the 31 patients and further 4 (12.9 %) patients had an increased amount of total urinary protein. In none of the patients, microhematuria or increased excretion of immunoglobulins, indicated by high-molecular-weight proteinuria, was found. Organ involvement and concomitant medication was similar to other patients in the DNSS registry as previously published [3].

**Fig. 2:**
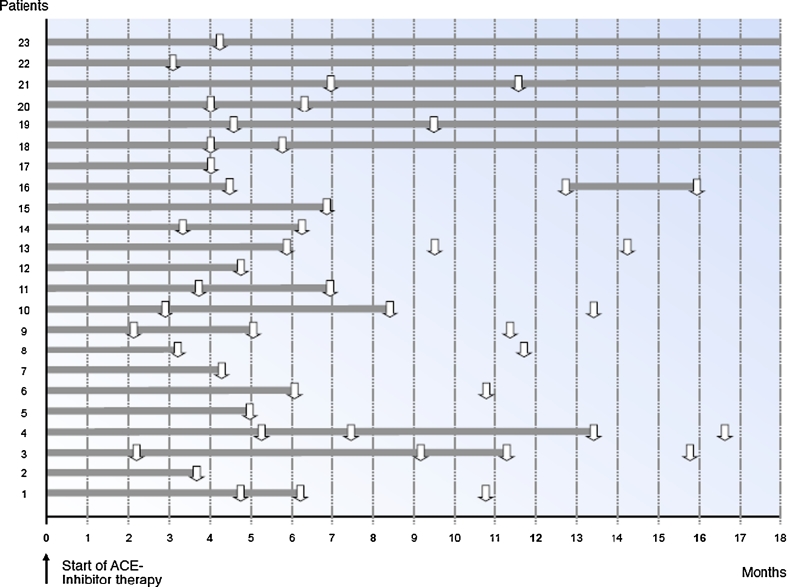
Duration of pathologic microproteinuria The *x*-axis indicates follow-up in months, the *y*-axis indicates individual patients (*n* = 23). The *grey bars* indicate the duration of pathological microproteinuria. *Arrows* indicate time points of urine analysis

Twenty-three of the 31 patients received ACE inhibitor treatment. During an observation period with a median of 12 months (range 3–18 months), a normalization of proteinuria was observed in 17 (73.9 %) of the 23 patients treated. In the untreated control group, an improvement was observed only in 2/8 patients (25 %, *p* = 0.014). The duration of pathologic urinary microproteinuria in individual patients is shown in Fig. 2.

Furthermore, in several patients with pathological albuminuria and/or elevated total protein excretion, an improvement was observed during ACE inhibitor therapy. Two out of 7 patients with albuminuria and 2 of the initially 3 patients with elevated total protein excretion showed a normalization of proteinuria associated with ACE inhibitor therapy. None of the treated patients developed hypotension, renal insufficiency or renal crisis.

The improvement of pathological protein excretion, when correlated with clinical characteristics, was found to be associated with a shorter disease duration, recently diagnosed proteinuria, low inflammatory activity and lack of additional immunosuppressive medication. However, due to the low number of patients, these correlations did not reach statistical significance.

## Discussion

Renal involvement in SSc can occur either as a rare complication of acute kidney failure, well known as scleroderma renal crisis, or as chronic progressive renal failure with slow deterioration of kidney function. End-stage renal disease in scleroderma is rare. In a recent study by Siva et al. [21], the analysis of a large registry of ESRD patients in Australia and New Zealand revealed a prevalence of 0.3 % for scleroderma patients. Median survival of these patients was significantly shorter compared to non-scleroderma ESRD patients.

A central, early component of the vascular pathophysiology in SSc is endothelial injury. Pronounced subendothelial thickening with deposition of mucinous material is observed in the interlobular arteries finally leading to a collapse of tubuli due to postarteriolar ischaemia. It is assumed that the loss of capillaries, which can be observed in affected organs, is due to an increase in inflammation, angiostatic factors and programmed cell death. In the kidney, endothelial damage is known to accelerate glomerular leakage finally leading to proteinuria.

Microalbuminuria is commonly used as an indicator of early kidney damage, and the detection of increased albumin and/or total protein excretion is associated with poorer renal and cardiovascular prognosis [12, 22–24]. Furthermore, albuminuria has been shown to be a marker for systemic vasculopathy in patients with cardiovascular disease [9]. Therefore, proteinuria as an early preclinical marker of renal pathology might also serve as a surrogate marker for the severity of renal vascular pathology and for prognosis in SSc [4]. This assumption is supported by recent studies describing proteinuria as an independent prognostic factor for survival in SSc [15, 16, 25]. Microalbuminuria is a hallmark of early diabetic nephropathy. Although diabetic nephropathy is caused by a different pathomechanism affecting primarily glomerular capillaries, it is clear from large trials that renal outcome is improved when treating patients with ACE inhibitors [26, 27]. The presence of proteinuria alone is capable of increasing the risk of a cardiovascular event to the same extent as a prior myocardial infarction [28]. Therefore, lowering proteinuria in SSc by ACE inhibitor treatment may have the same beneficial effect on the incidence of cardiovascular events in SSc patients.

In one of the few studies investigating chronic renal involvement in SSc in more detail, Steen et al. [2] found that 32 % of patients with diffuse SSc had abnormal renal function and/or proteinuria. However, in their study, proteinuria (defined as proteinuria(3 or 4+) on 2 occasions on dipstick analysis or >250 mg/24 h) was mostly attributed to the use of D-penicillamine or to other medical comorbidities.

In 2008, Seiberlich et al. [20] performed a detailed analysis of proteinuria in 80 patients with SSc with abnormalities being present in 31.3 % of patients, confirming results of Steen et al. In addition to standard methods of urinary protein determination, they used urine microgradient gelelectrophoresis, which is among the most sensitive methods for the detection of proteinuria. They found a significant association between mixed proteinuria and the diffuse type of systemic sclerosis, gastrointestinal involvement and increased systolic blood pressure. A correlation between albuminuria and increased systolic blood pressure, as well as between albuminuria and longer illness duration (4 year period) was also observed.

It is well established that the use of ACE inhibitors in diabetes and hypertension leads to an improvement of proteinuria, renal function and the risk of death and dialysis [17–19]. In line with these studies, we observed a normalization of urinary protein excretion in 74 % of SSc patients treated by ACE inhibitors. It could also be demonstrated that the normalization of microelectrophoresis was accompanied by a decrease in albuminuria and total urinary protein. The response to ACE inhibitor therapy was less pronounced in patients with an elevated erythrocyte sedimentation rate (ESR) and in those on immunosuppressive therapy reflecting a more intense inflammatory phase of the disease. Of interest, in the recently published large QUINS-Trial, no effect on 24-h total protein excretion was found after prolonged therapy with Quinapril [29]. However, this may well be due to the fact that only a comparison of total urinary protein excretion between the verum and the placebo group was made (being on average within normal limits in both groups).

The beneficial effect of ACE inhibition in this study might be attributed to (1) the anti-proteinuric effect of the ACE inhibitors by lowering intraglomerular pressure via reduction of angiotensin II levels and (2) the inhibition of the transforming growth factor-β (TGF-β) signalling cascade, whereby ACE inhibitors possess additional direct anti-fibrotic and anti-inflammatory potential [17, 30, 31]. This aspect may be of particular relevance for the treatment for renal disease in SSc where TGF-β has been shown to play a major pathogenetic role [32].

Previously, it had been stated that prophylactic use of ACE inhibition in SSc may not protect against renal crisis and may be even associated with worse outcomes [5, 6, 33]. On the other hand, the use of ACE inhibitors in scleroderma renal crisis was reported to be beneficial [34]. In patients followed in our study, no adverse events associated with ACE inhibitor therapy were observed.

In conclusion, this study shows for the first time that in SSc patients, ACE inhibitor therapy can lead to a normalization of urine protein excretion, a surrogate marker of renal prognosis. A controlled trial will be required to confirm these promising findings.
